# Biosecurity measures to control hepatitis E virus on European pig farms

**DOI:** 10.3389/fvets.2024.1328284

**Published:** 2024-02-14

**Authors:** Tamino Dubbert, Marina Meester, Richard Piers Smith, Tijs J. Tobias, Ilaria Di Bartolo, Reimar Johne, Enrico Pavoni, Gergana Krumova-Valcheva, Elena Lucia Sassu, Christopher Prigge, Giuseppe Aprea, Hannah May, Nadine Althof, Giovanni Ianiro, Jacek Żmudzki, Albena Dimitrova, Giovanni Loris Alborali, Daniela D'Angelantonio, Silvia Scattolini, Noemi Battistelli, Elke Burow

**Affiliations:** ^1^Department of Biological Safety, German Federal Institute for Risk Assessment (BfR), Berlin, Germany; ^2^Department of Population Health Sciences, Faculty of Veterinary Medicine, Utrecht University (UU), Utrecht, Netherlands; ^3^Department of Epidemiological Sciences, Animal and Plant Health Agency (APHA) - Weybridge, Surrey, United Kingdom; ^4^Department of Food Safety, Nutrition and Veterinary Public Health, Istituto Superiore di Sanità (ISS), Rome, Italy; ^5^Food Safety Department, Experimental Zooprophylactic Institute of Lombardy and Emilia Romagna (IZSLER), Brescia, Italy; ^6^National Food Safety Center, National Diagnostic and Research Veterinary Medical Institute (NDRVMI), Sofia, Bulgaria; ^7^Institute for Veterinary Disease Control, Austrian Agency for Health and Food Safety (AGES), Mödling, Austria; ^8^Department of Food Safety, Experimental Zooprophylactic Institute of Abruzzo and Molise ‘G. Caporale' (IZS), Teramo, Italy; ^9^Department of Swine Diseases, National Veterinary Research Institute (PIWet), Puławy, Poland; ^10^Department for Rural Development and Agriculture, Ministry of Agriculture, Environment and Climate Protection of the State of Brandenburg (MLUK), Potsdam, Germany

**Keywords:** hepatitis-E-virus, HEV, biosecurity measures, pig production, BIOPIGEE, One Health, OH

## Abstract

Hepatitis E virus (HEV) genotype 3 is a prevalent zoonotic pathogen in European pig farms, posing a significant public health risk primarily through the foodborne route. The study aimed to identify effective biosecurity measures for controlling HEV transmission on pig farms, addressing a critical gap in current knowledge. Utilizing a cross-sectional design, fecal samples from gilts, dry sows, and fatteners were collected on 231 pig farms of all farm types across nine European countries. Real-time RT-PCR was employed to test these samples for HEV. Simultaneously, a comprehensive biosecurity questionnaire captured data on various potential measures to control HEV. The dependent variable was HEV risk, categorized as lower or higher based on the percentage of positive pooled fecal samples on each farm (25% cut-off). The data were analyzed using generalized linear models (one for finisher samples and one for all samples) with a logit link function with country and farm type as *a priori* fixed factors. The results of the final multivariable models identified key biosecurity measures associated with lower HEV risk, which were the use of a hygienogram in the breeding (OR: 0.06, *p* = 0.001) and/or fattening area after cleaning (OR: 0.21, *p* = 0.019), the presence of a quarantine area (OR: 0.29, *p* = 0.025), testing and/or treating purchased feed against Salmonella (OR: 0.35, *p* = 0.021), the presence of other livestock species on the farm, and having five or fewer persons in charge of the pigs. Contrary to expectations, some biosecurity measures were associated with higher HEV risk, e.g., downtime of 3 days or longer after cleaning in the fattening area (OR: 3.49, *p* = 0.005) or mandatory handwashing for farm personnel when changing barn sections (OR: 3.4, *p* = 0.026). This novel study unveils critical insights into biosecurity measures effective in controlling HEV on European pig farms. The identification of both protective and risk-associated measures contributes to improving strategies for managing HEV and underscores the complexity of biosecurity in pig farming.

## 1 Introduction

The hepatitis E virus (HEV) is the causative agent of acute and chronic hepatitis in humans (1). Worldwide, HEV infections account for an estimated 3.3 million symptomatic cases and 44,000 deaths per year (2). Depending on the involved HEV genotype, severe disease symptoms and deaths are mainly observed in pregnant women, organ transplant recipients, patients with pre-existing liver disease and immunosuppressed patients (1). However, serological population surveys indicate that mild and subclinical HEV infections are more common (3, 4).

The HEV genotypes 1 and 2 (HEV-1 and HEV-2) only infect humans and are waterborne, causing large outbreaks in African and Asian countries with poor sanitary conditions (5, 6). In contrast, HEV-3 and HEV-4 genotypes are zoonotic, with HEV-3 being common in North America and Europe (5) and HEV-4 mainly confined to Asia (6). For a European context, HEV-3 is of most interest. The main reservoirs for HEV-3 are pigs and wild boars, although the virus has been detected in other animals like deer and rabbits (7, 8). While asymptomatic in pigs and wild boar, the disease can sporadically be acute and lethal in humans (8, 9). Transmission of HEV-3 to humans is considered to be foodborne or by direct contact with infected animals and can often be traced back to the consumption of raw or undercooked contaminated pork and wild boar meat, especially liver (5, 7, 10, 11).

Although HEV is estimated to be present on many pig farms in Europe, there are differences in HEV seroprevalence between farms and countries, ranging from 65 to 100% at the farm-level and from 20 to 93% at the animal-level (7, 12, 13). Seroprevalence increases with the age of animals up to 100% in adult pigs, proving frequent exposure to the virus (14, 15). After introduction, HEV has been shown to persist on farms for multiple years (16, 17). To reduce the burden to public health, risk mitigation for HEV infection should not only occur at the slaughterhouse, but also in primary production, i.e., at farm level. Biosecurity measures are commonly defined as measures to prevent the introduction of pathogens into and their spread within a farm. However, published knowledge on effective control strategies for HEV in pig farms is very limited (18).

Several studies investigated the associations of risk factors with different HEV outcomes (19). Risk of HEV positive livers in slaughter-age pigs was increased by a large variation in age of pigs sent to slaughter, a high cross-fostering rate at farrowing, use of boots that were not specific for swine production, drinking water supply from a spring or a well drilled <50 m deep, and maternal genetic background of the pigs (20). Risk of higher HEV seroprevalence in slaughter-age pigs was increased by a down period of <4 days in the nursery, a shorter distance between pit manure and slatted flooring in fattening premises, mingling of pigs from different premises between farrowing and nursery stages, and pen sizes of 16 or more pigs/pen in nursery rooms, while gilts' acclimatization via distribution of placenta and feces from sows decreased risk of higher HEV seroprevalence (20). Risk factors associated with greater HEV prevalence in pigs are extensive farming, absence of a sanitary ford, no quarantine period, and contact with other domestic species (21). Risk factors associated with higher presence of anti-HEV antibodies at farm were not performing disinfection after cleaning, and mixed drinking water systems, i.e., with stagnant and running water (22). In a previous study of three Japanese farms, the one that did not mingle pigs during weaning had the lowest HEV seroprevalence (23). A Canadian study observed widespread HEV infections in weaned piglets coming from multiple suppliers (24). This study investigated the natural course of infection during the grow-out period in a simulated farm setting, and found that almost all of the pigs shed HEV on at least one occasion. However, more research is needed to strengthen evidence and to prioritize measures for the control of HEV.

This epidemiological risk factor study investigated, in a population of different European pig farms, the associations of HEV with a large number of biosecurity measures based upon previously published literature and expert opinion. HEV risk of farms was estimated based on the number of HEV-positive fecal samples using a novel protocol of detection. The results should help to improve evidence-based farm biosecurity and control HEV in pig farming.

## 2 Materials and methods

### 2.1 Study design and sample description

**Figure 1:**
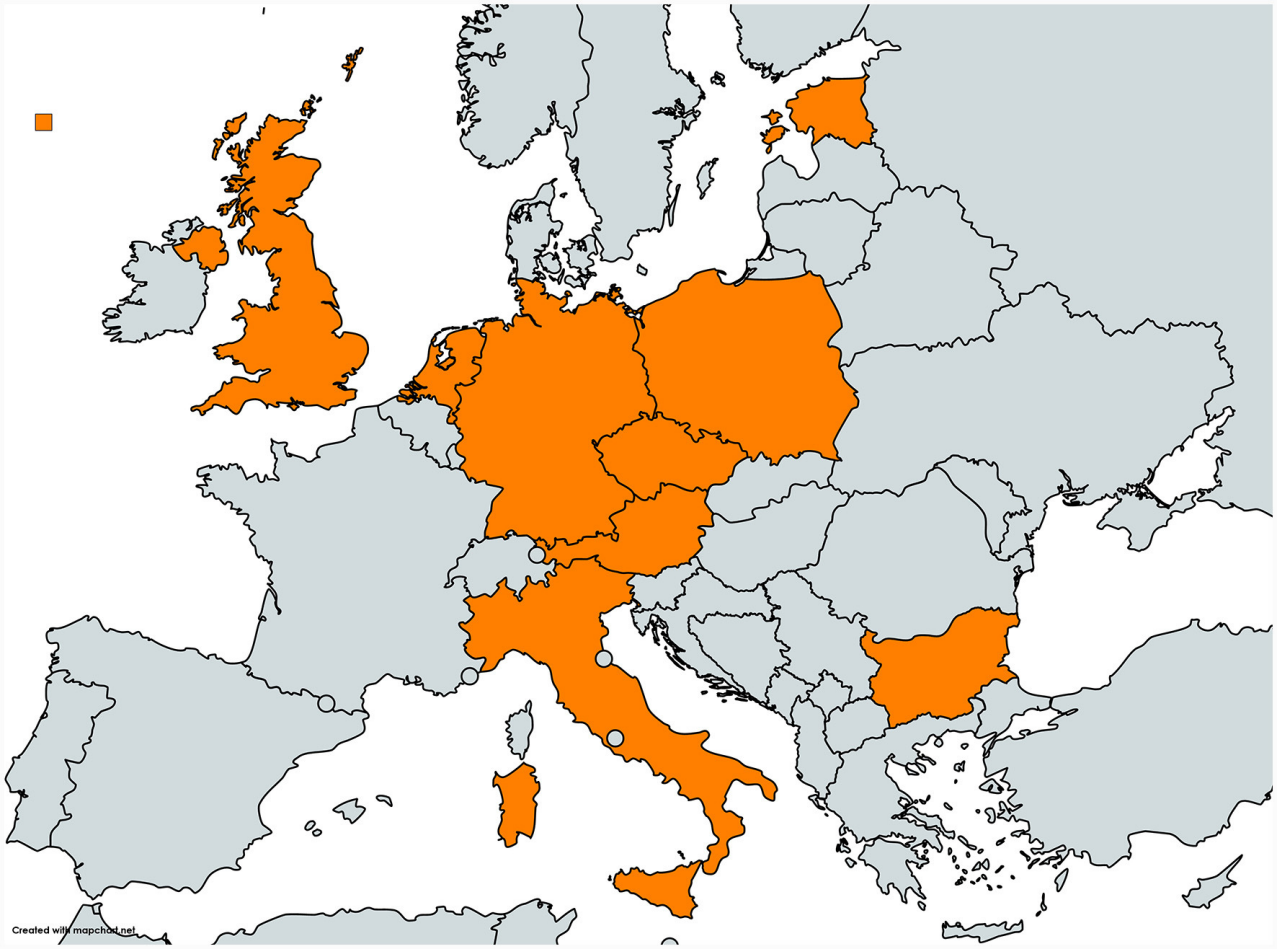
Map of the nine European countries Austria, Bulgaria, Czech Republic, Germany, Estonia, Italy, the Netherlands, Poland and the United Kingdom participating in the OHEJP BIOPIGEE project (created with mapchart.net and shared under CC BY-SA 4.0).

The study design is fully described in the paper focused on *Salmonella* results from the same study group (25). Briefly, this cross-sectional study aimed to include 30 farms of all main production types from each of the nine participating European countries: Austria (AT), Bulgaria (BG), Czech Republic (CZ), Germany (DE), Estonia (EE), Italy (IT), the Netherlands (NL), Poland (PL) and the United Kingdom (UK, Figure 1). Although farm selection was by convenience, it was aimed to include a farm population representative for each country in terms of size and types of pig farms. Farms were excluded if they were small holdings, kept some pigs of a specific type indoors and others of the same type outdoors, as well as nucleus/multiplier herds or specific pathogen free (SPF) herds. Nucleus/multiplier and SPF herds were excluded, since they are few in number and difficult for visitors to access, which may lead to selection bias.

### 2.2 Data collection

Data were collected from recruited farms between July 2020 and October 2021.

#### 2.2.1 Questionnaire

The questionnaire applied in this study is described in detail elsewhere (26). In brief, it included 10 questions on farm characteristics and 56 questions focusing on biosecurity practices related to indoor pig production. Questions could be answered by choosing one of the answer options (categorical) or providing a number. The basis for the selection of questions and related biosecurity measures was their relevance to reduce HEV and *Salmonella* occurrence in pig production (as stated by the OHEJP BIOPIGEE project) according to peer-reviewed articles and experts' opinion (26–28). An additional criterion for including biosecurity measures to the questionnaire was their practicality, i.e., measures should be actively changeable by the farmer in a reasonable period of time (e.g., questions about neighbor farms, locations or fundamental farm constructions were excluded). The questionnaire was translated into the languages of the participating European countries and set up in an electronic survey tool (keyingress/mobilingress, Ingress Health GmbH, Germany).

The questionnaire was completed on-farm by the farmer in collaboration with the staff from the BIOPIGEE partner institutes during the visit to collect fecal samples. Part of the interviews and completion of questionnaires had to be carried out over phone calls, separately from the sampling, due to SARS-CoV-2 pandemic restrictions. Interviewers and interviewees had no knowledge of the HEV status of the farms.

#### 2.2.2 HEV detection

##### 2.2.2.1 Sample collection

The prevalence of HEV on farms had been estimated in only a small number of participating countries prior to the study commencing (12) and could therefore not be used to calculate sample size. The sample size was instead based on the ability to detect both HEV and *Salmonella* (25), even if present at a relatively low prevalence. To accommodate that, pooled samples were used. Moreover, three different categories of pigs (gilts, dry sows and fatteners) were to be sampled, to maximize the chance of detecting both HEV and *Salmonella* on the farms, based upon previous studies and expert opinion within the BIOPIGEE consortium (29, 30).

The optimal number of fecal samples to stratify between farms with higher and lower risk status of HEV was determined after discussions within the project team, which included HEV experts. Twenty pooled fecal samples per farm (10 individual samples per pooled sample) were determined as the optimal number. This provided sufficient sensitivity to detect at least one positive sample even if the within-herd prevalence was as low as 2% and would estimate an expected farm prevalence of 10% (31, 32) with 5.5% variance and 95% confidence (33).

Each individual sample contained 10 g of fresh feces, preferably collected immediately after defecation. Additionally, when more pens were present in the farm, as many pens as possible were sampled covering a uniform spatial distribution. The ratio of samples collected for three types of pigs (fattener/gilt/dry sow) was according to the farm type: 50%/40%/10% on farrow-to-finish farms, 0%/80%/20% on breeding farms, and 100%/0%/0% on fattening farms. For example, breeding farms were to have 16 gilt and four dry sow pooled samples collected. Fecal samples were collected from fattening pigs before the age of slaughter (~4–6 months old).

Farms from NL in this study (*n* = 20) were sampled originally for a different study. An average of eight batches of finishing pigs delivered to slaughter for each farm were sampled by blood collection of five to 12 random pigs per batch. Serum was tested individually for HEV antibodies and tested pooled per batch for viremia [for study design see Meester et al. (15)]. The NL samples were collected from January to August 2019. Between 12 and 173 pigs per farm were sampled (median 51, mean 64.8). Each farm had between 4 and 21 batches (median 8.5, mean 10.2). Samples were pooled at batch level and tested for HEV RNA by real-time RT-PCR.

##### 2.2.2.2 Sample testing

Samples were transported to testing laboratories in cooling boxes not to exceed 8°C and finally stored at −20°C until testing for HEV. After thawing, stools were diluted 1:10 (w/v) in sterile RNase-free water or phosphate-buffered saline and were clarified by low-speed centrifugation at 10% (w/v). Before RNA extraction, the fecal supernatants were artificially spiked with a process control virus, which consisted of mengovirus (34), murine norovirus (35), feline calicivirus (36), or bacteriophage MS2 (37). Viral RNA was extracted from 100 μl of supernatant by commercial silica-based kits different for each country (Qiamp Viral mini kit, Qiagen; MiniMag kit, Biomerieux; EMAG kit, Biomerieux; BioExtract^®^ SuperBall^®^, BioSellal; MagMAX Viral/Pathogen Nucleic Acid Isolation Kit, Thermofisher) and eluted in a total volume of 100 μl elution buffer. RNA was stored at −80°C or immediately analyzed.

The RNA of the process control virus used for spiking fecal samples was analyzed by a different real-time RT-PCR (34, 38, 39). The resulting recovery rate was estimated by the comparative cycle threshold method (40). A recovery rate >1% was considered suitable for the subsequent amplification analyses of the HEV target virus (34).

For HEV RNA detection, a broad range real-time RT-PCR was conducted as described previously (41). All participating laboratories used 5 μl of RNA to prepare a reaction mix with a total volume of 25 μl using different real-time RT-PCR kits (QuantiTect Probe RT-PCR Kit, Qiagen; RNA UltraSense™ One-Step qRT-PCR System, Thermofisher Scientific) (42, 43). Each real-time RT-PCR included RNA from fecal samples, negative extraction controls, water control (NTC), and positive target RNA control for each run.

To enable comparability of results generated by the different extraction and amplification protocols, the limit of detection (LOD) of the used method was determined by each laboratory prior to participation in the study. This was performed by testing two-fold dilution series of HEV (1st WHO International Standard for Hepatitis E virus RNA, PEI code 6329/10, Paul-Ehrlich Institute, Germany) (44) in an HEV-negative pig stool sample. By this, the participating laboratories determined LODs between 87.2 × 10^3^ and 10.9 × 10^3^ HEV genome copies/g stool.

#### 2.2.3 Statistical analysis

##### 2.2.3.1 HEV risk categorization

The aim of this study was to find evidence for effective biosecurity measures to control HEV by comparing biosecurity measures present on those farms with a lower percentage of HEV-positive samples against those farms with a higher percentage, using a multivariable risk factor analysis. The hypothesis was that farms performing effective biosecurity measures were at a lower risk of having a higher percentage of HEV-positive samples compared to farms that were not performing effective biosecurity measures. The cut-off for this binary HEV risk categorization of farms was chosen to be 25%, meaning farms with equal to or more than 25% HEV-positive samples were categorized as higher risk. The choice for this cut-off value was made by the study team only after the inspection of the distribution of HEV-positive samples of all farms (see Section 3.3).

##### 2.2.3.2 Questionnaire data

Questionnaire data was cleaned and potentially incorrect information checked with the farmers or the local project team. Missing answers were coded as “missing” or “not applicable” where it made sense to allow this information to be retained in the model. Also, to improve model fit, merging of levels with 10 or fewer observations with other levels of the same variable was explored when meaningful. Continuous variables were plotted to assess possible trends or groupings and subsequently categorized.

##### 2.2.3.3 Data analysis

Descriptive analyses were performed and results presented. The distribution of positive fecal samples was investigated at sample level as a basis to determine a meaningful cut-off value for risk categorization of farms. Country data pertaining to HEV-status or HEV-risk is only shown in an anonymized form (Country A–I).

The outcome variable was the HEV risk of the farms based on real-time RT-PCR results from fecal sample analysis. For the multivariable statistical analysis, the outcome was dichotomized (0 = percentage of HEV-positive fecal samples below defined cut-off, 1 = percentage of HEV-positive fecal samples above defined cut-off). All associations between outcome and independent variables were investigated in generalized linear models with a logit link function (glm function) and country and farm type as *a priori* fixed factors. Variables that were associated in preliminary univariable analyses with HEV risk (*p* < 0.25) were selected for risk factor modeling. Variables were excluded from the selection when failure or success were predicted perfectly (e.g., 0 observations in one cell), or when farms almost unanimously confirmed or denied the variable/measure (e.g., ≤ 10 farms affirming or denying). A Cramer's *V* correlation matrix (char_cor_vars function) was produced to identify strong correlations between variables. If a correlation of ≥0.8 between two independent variables was observed, the one with the stronger correlation with the outcome was retained for risk factor modeling. If the variables were equally correlated with the outcome, one was chosen by the study team.

Risk factor modeling was performed using forward-stepwise logistic regression. In subsequent steps, those variables that most improved the fit of the model [lowest Akaike Information Criterion (AIC)] were included until a step was reached where no further variables were significant and could improve the model fit. Sensitivity analyses were performed by excluding NL farms from the model. Data cleaning and recoding was performed with SAS (version 15, Statistical Analysis System, RRID:SCR_008567) and R (version 4.1.2, R Project for Statistical Computing, RRID:SCR_001905). All statistical analyses were performed with R.

## 3 Results

### 3.1 Farm population

Questionnaire and HEV sample data were available for 231 farms. Of these, 119 (51.5%) were farrow-to-finish farms, 39 (16.9%) were breeding farms, and 73 (31.6%) were fattening farms. Only six (2.6%) farms were outdoor farms. Data from sampled countries was available for between three (EE) and 47 (IT) farms (Table 1).

**Table 1:** Farm population according to country and farm type.

Country^*^	**Farrow-to-finish**	**Breeding**	**Fattening**	**Indoor**	**Outdoor**	**Total**
AT	14	4	2	20		20
BG	32	1		33		33
CZ	23	3	4	30		30
DE	9	9	12	30		30
EE	2		1	3		3
IT	9	17	21	45	2	47
NL	7		13	20		20
PL	14	1	15	30		30
UK	9	4	5	14	4	18
Total	119	39	73	225	6	231
% of total	51.5%	16.9%	31.6%	97.4%	2.6%	100%

* Austria (AT), Bulgaria (BG), Czech Republic (CZ), Germany (DE), Estonia (EE), Italy (IT), the Netherlands (NL), Poland (PL), and the United Kingdom (UK).

### 3.2 HEV results by farm

**Figure 2:**
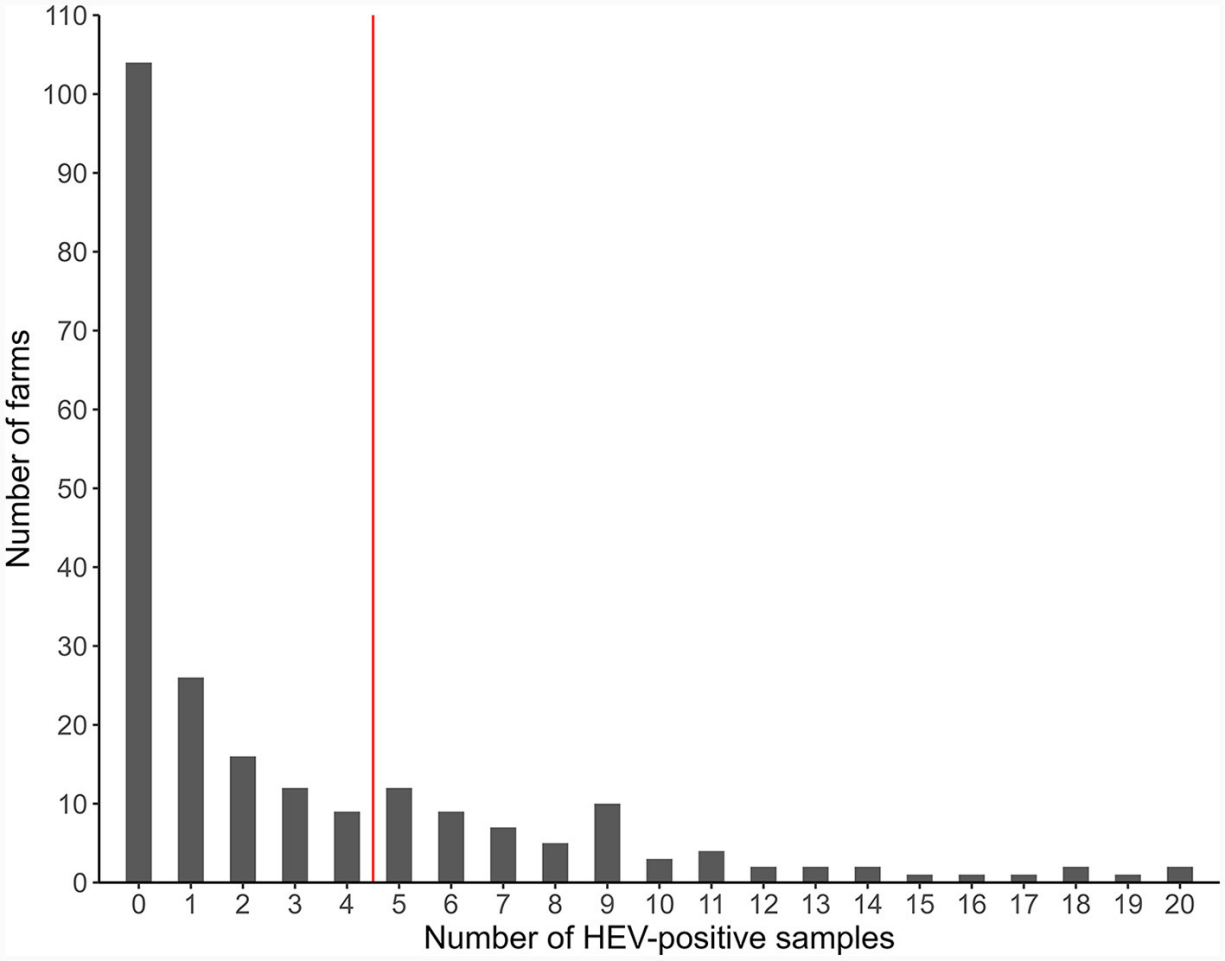
Distribution of the farms according to the number of HEV-positive pooled samples (231 pig farms, Europe, 2020).

**Figure 3:**
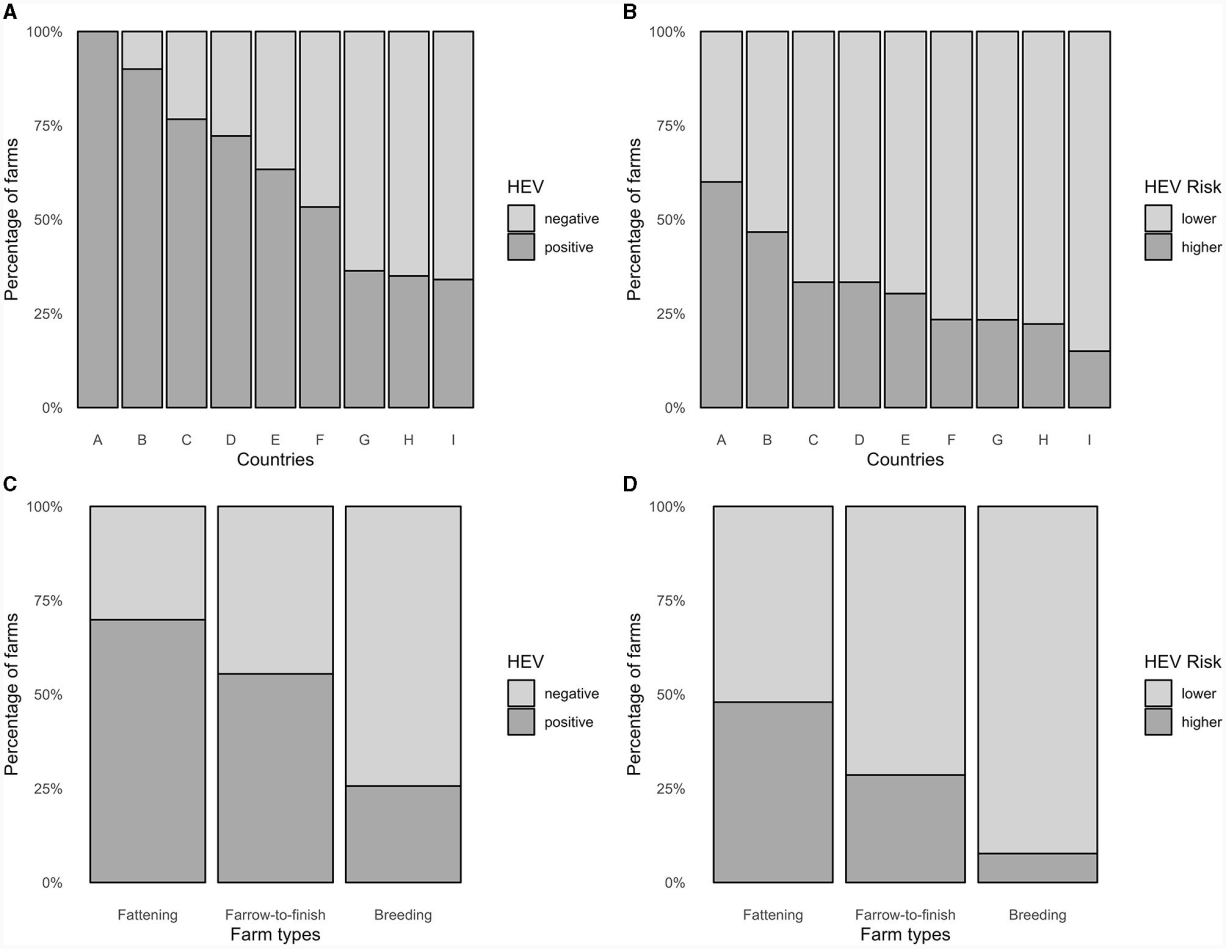
Distribution in percentages of **(A)** HEV-positive farms and **(B)** HEV-higher risk farms according to country, and **(C)** HEV-positive farms and **(D)** HEV-higher risk farms according to farm type (231 pig farms, Europe, 2020).

Of the 4,389 samples from pigs of all production stages, 718 (16.4%) were HEV-positive. Of the 231 farms, 127 (55%) had at least one positive sample (Figures 2, 3A). Fattening farms had the highest mean average percentage of positive samples (29.2%), followed by farrow-to-finish (13.0%) and breeding farms (4.5%). Accordingly, fattening farms had the highest percentage of HEV-positive farms (69.9%), followed by farrow-to-finish (55.5%), and breeding farms (25.6%, Figure 3C). Two of the six outdoor farms were positive (33.3%), with a mean average of 5.9% positive samples among all and 17.9% among only the positive outdoor farms (Supplementary Tables 1–4).

HEV-positive fattener samples from NL fattener farms were comparable to positive fattener samples from fattener farms of the other countries (29.6 vs. 29.2%). However, fattener samples from NL farrow-to-finish farms were more frequently positive than fattener samples from farrow-to-finish farms of the other countries (39.7 vs. 22.0%).

### 3.3 HEV risk

The cut-off for HEV risk categorization was set at 25%, i.e., farms with 25% or more positive samples would be in the higher HEV risk category. This assessment was based of the distribution of positive samples of all farms and in order to produce useful populations for the analysis (Figure 2). Of the 231 farms, 72 (31.2%) were categorized as being at higher risk for HEV, based on all samples. Higher-risk farms were most common among fattening farms followed by farrow-to-finish and breeding farms, with 35 (47.9%), 34 (28.6%), and 4 (7.7%) farms of each type being categorized as higher risk (Figures 3B, D, Supplementary Table 5).

**Figure 4:**
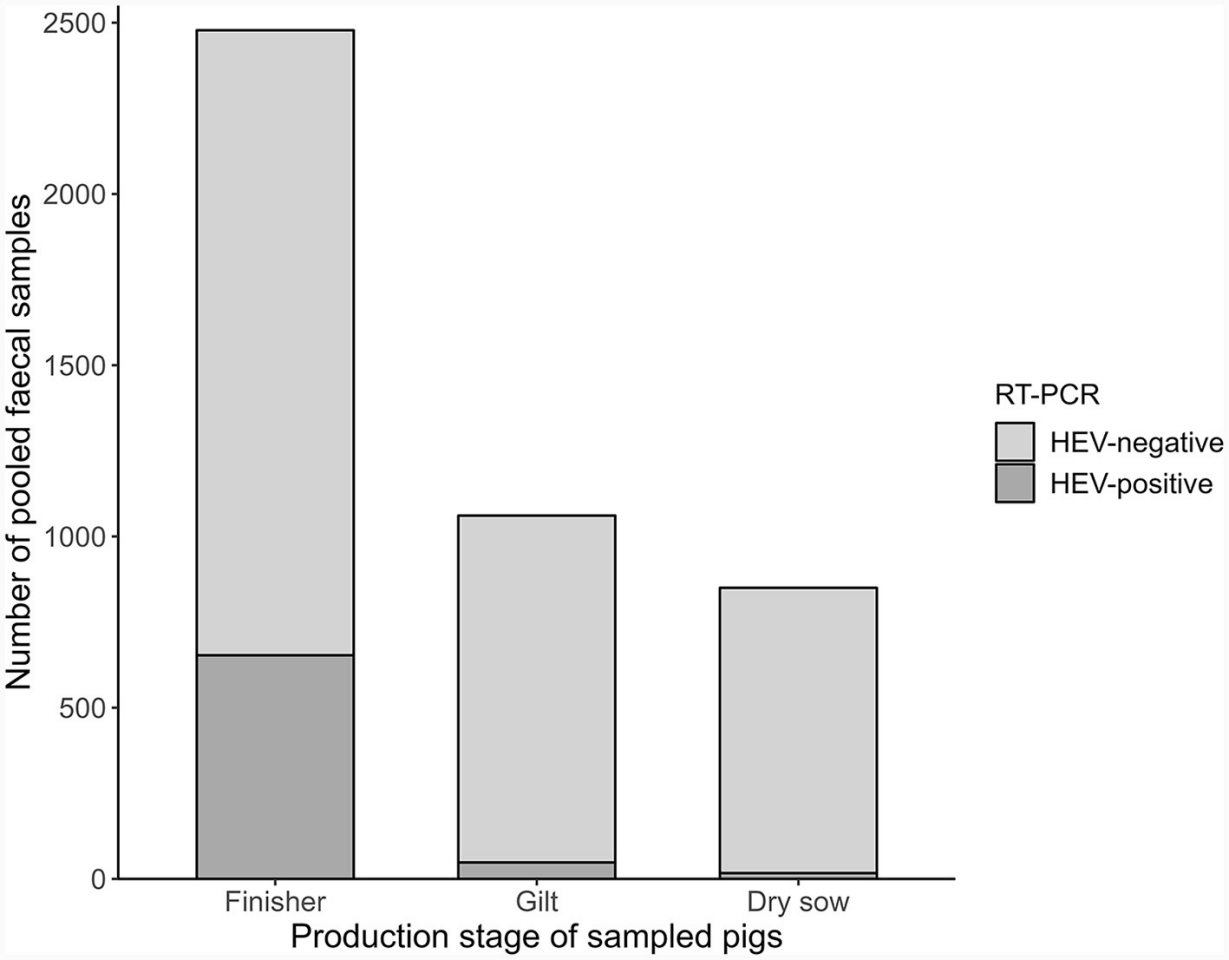
Distribution of HEV-positive samples tested by real-time RT-PCR according to pigs sampled (Europe, 2020).

Samples from fattener pigs were significantly more likely to be HEV-positive than samples from gilts or dry sows (OR: 10.2, 95% CI: 7.87–13.34, *p* < 0.001, Figure 4). Therefore, the study team decided to perform a second risk factor model procedure only with those farms that provided fattener samples and only using the fattener samples for risk categorization. Here, the same cut-off of 25% was used. Of the 188 farms with fattener samples, 77 (41%) were categorized as being at higher HEV risk. Four farrow-to-finish farms had no fatteners at the time of sampling. A chi-squared test showed that farms with 10 or fewer fattener samples collected, such as the farrow-to-finish farms and some farms from NL, were not significantly more or less likely to be in the lower risk category than farms with more than 10 fattener samples (OR: 1.32, 95% CI: 0.69–2.5, *p* = 0.373).

### 3.4 Univariable results

In univariable regression analyses adjusted for country and farm type, nine variables were significantly associated with HEV risk based on all samples and 10 variables were significantly associated with HEV risk based on fattener samples only (Supplementary Tables 6, 7).

### 3.5 Risk factor model—HEV positive samples from all pigs

For the risk factor model based on all pig samples, 65 variables remained after the univariable regression stage, 15 of them were subsequently excluded due to high correlation. The stepwise procedure resulted in a final model with nine variables retained, including country and farm type. Lower odds of being at higher risk of HEV, based on all samples, were associated with fewer persons working with the pigs, use of a hygienogram as part of the cleaning procedure in the breeding area, and presence of a quarantine area. Higher odds of being at higher risk of HEV, based on all samples, were associated with downtime of at least 3 days in the fattening area, internal people (i.e., staff) washing hands between different barn sections, no disposable gloves worn and/or hands washed and disinfected when manipulating carcasses, and wild birds having access to the barns (Table 2). The model was estimated to have explained 27.2% of the variation in the outcome (McFadden's pseudo-*R*^2^ = 0.272).

**Table 2:** Multivariable logistic regression model with HEV risk based on all pig samples adjusted for country and farm type.

**Question**	**Levels**	**Higher HEV risk farms *n* (%)**	**OR**	**95% CI [LL, UL]**	***p*-value**
Number of people in charge of the pigs	6+	24 (38.7)	1		
	1–5	48 (28.4)	0.1	0.03, 0.32	<0.001
Is an efficacy check with a hygienogram part of the cleaning procedures in the breeding area?	No	31 (24.6)	1		
	Yes	5 (16.7)	0.06	0.01, 0.28	0.001
	N/A	36 (48)	10.22	0.02, 9,715.29	0.681
Is a quarantine area present at your farm?	No	16 (30.2)	1		
	Yes	22 (20)	0.29	0.09, 0.84	0.025
	N/A	34 (50)	0.7	0.06, 17.77	0.788
Is downtime of at least 3 days part of the cleaning procedure in the fattening area?	No	28 (28.6)	1		
	Yes	42 (40)	3.49	1.49, 8.56	0.005
	N/A	2 (7.1)	0.31	0.02, 4.18	0.369
Do internal people always have to wash hands between different barn sections?	No	52 (29.4)	1		
	Yes	17 (38.6)	3.4	1.18, 10.3	0.026
	N/A	3 (30)	0.25	0.03, 1.38	0.133
Are disposable gloves worn when manipulating carcasses and/or are hands washed and disinfected after manipulating carcasses?	No	8 (34.8)	1		
	Yes	64 (30.8)	8.91	1.56, 79.73	0.025
Do wild birds have access to the barns?	No	46 (30.3)	1		
	Yes	24 (33.3)	2.47	1.06, 5.98	0.040
	N/A	2 (28.6)	2.69	0.27, 22.04	0.361

The table presents the corrected odds ratios (OR), its 95% confidence intervals (CI), and p-values.

### 3.6 Risk factor model—HEV positive fattener samples

For the risk factor model based on fattener samples only, 54 variables remained after the univariate regression stage, 10 of them were subsequently excluded due to high correlation. The stepwise procedure resulted in a final model with seven variables retained, including country and farm type. Lower odds of being at higher risk of HEV, based on fattener samples only, were associated with fewer persons working with the pigs, testing and/or treating purchased pig feed against Salmonella contamination, use of a hygienogram as part of the cleaning procedure in the fattening area, presence of a quarantine area, and presence of other livestock species on the farm. Higher odds of being at higher risk of HEV, based on fattener samples only, were associated with purchasing pigs and wild birds having access to the barns (Table 3). The model was estimated to have explained 20.5% of the variation in the outcome (McFadden's pseudo-*R*^2^ = 0.205).

**Table 3:** Risk factor model with HEV risk based on fattener samples only and adjusted for country and farm type.

**Question**	**Levels**	**Higher HEV risk farms *n* (%)**	**OR**	**95% CI [LL, UL]**	***p*-value**
Number of people in charge of the pigs	6+	24 (51.1)	1		
	1–5	53 (37.6)	0.12	0.03, 0.41	0.002
Is purchased feed always tested for and/or treated against Salmonella contamination?	No	35 (48.6)	1		
	Yes	42 (36.2)	0.35	0.14, 0.84	0.021
Is an efficacy check with a hygienogram part of the cleaning procedures in the fattening area?	No	69 (43.7)	1		
	Yes	8 (26.7)	0.21	0.06, 0.75	0.019
Is a quarantine area present at your farm?	No	18 (48.6)	1		
	Yes	25 (30.1)	0.22	0.07, 0.65	0.007
	N/A	34 (50)	0.44	0.04, 11.53	0.548
Are pigs purchased?	No	20 (31.7)	1		
	Yes	57 (45.6)	3.21	1.31, 8.33	0.013
Are other livestock species present on the farm?	No	65 (43.9)	1		
	Yes	12 (30)	0.28	0.1, 0.75	0.013
Do wild birds have access to the barns?	No	49 (38.3)	1		
	Yes	26 (47.3)	2.5	1.1, 5.86	0.031
	N/A	2 (40)	2.66	0.24, 27.94	0.400

The table presents the corrected odds ratio (OR) estimates and its 95% confidence interval as well as the p-value estimate.

### 3.7 Sensitivity analysis

Since NL farms were sampled differently than farms from the other countries, sensitivity analyses were performed by excluding NL farms from the multivariate models (Supplementary Tables 8, 9). In the model based on all samples, excluding NL farms lead to the carcass handling variable losing its significance but did not lead to instability of the model overall or strong estimate changes. In the model based on fattener samples only, excluding NL farms had no effect on model stability and estimates stayed in the same range.

## 4 Discussion

Multivariate analyses revealed various biosecurity measures significantly associated with HEV risk on European pig farms. While there have been studies investigating infection dynamics of HEV on pig farms, only a few studies have investigated biosecurity measures in relation to HEV (20, 21, 45, 46). In the risk factor models, cleaning procedure steps, presence of a quarantine area, hygienic measures of farm personnel and the number of people in charge of the pigs were significantly associated with HEV risk. Some of the measures found were contrary to expectations.

This study represents the first investigation across Europe regarding the occurrence of HEV on pig farms. It reveals a mean HEV-RNA prevalence comparable to previous studies conducted within countries like in Switzerland (58.8%) (47), with some differences among countries, ranging between 35 and 100% (42, 43) (Figure 3A). The sample matrix and sampling scheme were determined by the BIOPIGEE study team as the most appropriate and cost-efficient method to detect and distinguish lower and higher risk farms for HEV, as well as for *Salmonella* (25). The two HEV outcomes accounted for the differences in HEV-positive samples from pigs at different production stages and possible effect modification by the low number of HEV-positive breeding farms in the first risk factor model. The biosecurity measures investigated were chosen based on their feasibility, expert opinion and evidence in published literature of their effectiveness to reduce HEV risk (and also *Salmonella*) on pig farms (28). Therefore, the comprehensive list of HEV biosecurity measures investigated in this study is unique and the first of its kind.

Utilizing hygienograms in the breeding and fattening areas was associated with lower HEV risk in the all-samples and fattener-samples model, respectively. Hygienograms, tests for bacterial growth, can be used to check the efficacy of cleaning procedures. In this study, farms using hygienograms were almost exclusively low-risk farrow-to-finish farms and applied hygienograms in all production stages. Those farms did significantly more cleaning steps in every production stage, compared to non-hygienogram using farms, and furthermore, were more likely to apply particular cleaning steps in most or all production stages, like dry cleaning, wet cleaning or downtime. The number of steps performed in cleaning procedures alone, however, was not associated with HEV risk (analysis not shown). In summary, this could mean that the use of hygienograms—although detecting bacteria—helps to identify and improve suboptimal cleaning, which subsequently reduces environmental load of HEV (48).

In both models the existence of a defined quarantine area was associated with lower HEV risk. This is in line with the study of Lopez-Lopez et al. (21), which found an association between the lack of a quarantine period and higher HEV risk. Quarantine areas are typically situated away from other farm buildings. Here, the health status of pigs coming to the farm, mainly gilts, is monitored and tested before they enter other farm buildings or getting mixed together with the herd. Additionally, sick pigs from the farm can be isolated here. Purchased gilts are typically between 4 and 6 months old, at which HEV is most prevalent (7, 49). Therefore, a quarantine period may be particularly effective for gilts to recover from infection and enter the pig herd HEV-negative.

Improving external biosecurity beginning with the quarantine area is highly relevant, since the frequent movement of pigs between farms is a risk factor and considered one of the main drivers of disease spread in modern specialized pig farming (50). And indeed, purchasing pigs was associated independently with higher HEV risk based on fattener samples. In this study, farms purchasing pigs were more likely to have a quarantine area compared to farms that did not buy pigs (excluding farms that had no quarantine area due to the production system, i.e., fattening farms). This and the fact that both variables—the quarantine area and purchasing pigs—were in the same model, indicates that having a quarantine area is protective against HEV, even when these farms are not purchasing pigs, and likewise, that purchasing pigs is a true risk factor for HEV even when these farms have a quarantine area.

Testing or treating purchased pig feed against *Salmonella* was associated with lower HEV risk in the all-samples model. However, the risk of HEV transmission to pigs via contaminated feed is considered low (18, 19). An explanation could be that testing and/or treating purchased feed (an external biosecurity measure) is reflective of a generally heightened sense of biosecurity and more diligent efforts to prevent pathogen introduction to and spread within the farm.

Wild birds having access to the barns was associated with higher HEV risk in both models. Although this is an example of poor biosecurity and a known risk factor for *Salmonella* (51), no specific risks regarding wild birds and HEV have been highlighted in previous studies. However, one study reported a natural infection of wild birds with mammalian HEV (genotype 4) in a wildlife center in China (52), indicating the possibility that wild birds may be able to (re-)introduce HEV into pig herds.

The presence of other livestock species on farm was associated with lower HEV risk based on fattener samples only. This small group of farms that had (one or multiple) other livestock species present was also significantly more often taking care of pigs with fewer people. This could indicate that these farms were smaller, less commercial and possibly had lower pathogen infectious pressure than bigger and more specialized farms. Additionally, there was no association between HEV risk based on fattener samples only and any specific type of other livestock species present.

Counterintuitive findings can be caused by higher risk farms having (recently) implemented measures of which their protective effect is not yet measurable or counteracted by insufficient biosecurity practices at other points on the farm. Estimates of actually protective measures would be biased and appear as not significant or even as risk factors, which could explain that fattening area downtime and cross barn section hygiene routines were associated with higher HEV risk in the all-samples model. Similarly, hygienic precautions when handling carcasses, a measure confirmed by a large majority of farms, was also associated with higher HEV risk in the same model. Notably, this particular risk effect disappeared after excluding farms from NL and therefore may have been caused by NL farms being more frequently and possibly unfairly categorized as higher risk.

Higher HEV risk was associated with fattening farms and fattening area variables, while the opposite was true for breeding farms (i.e., breeding farms and breeding area variables were associated with lower HEV risk). This HEV risk difference between farm types and related measures is reflective of the infection dynamics of HEV, as the infection peak commonly occurs during fattening (19, 53). Although varying efforts in biosecurity may play a role, fattening farms did not differ significantly from breeding farms in their implementation of various cleaning procedure steps (analysis not shown). While fattening farms need to prioritize control of on-farm spread and environmental HEV load, they rely on the healthiness of the pigs which they receive from farms earlier in the production chain.

This study has several limitations. Farms were a convenience sample, but efforts were made to select farms that were representative of pig farming within each participating country. Additionally, the HEV status of the farms was unknown before sampling which may have limited selection bias.

The method of HEV detection in feces is not yet standardized and differences in detection on a national level remain possible. However, efforts were made to harmonize limit of detection (LOD) protocols and results of a pre-study analysis showed comparable LODs among participating laboratories. All but one demonstrated successful detection even at the highest dilution of HEV-RNA. Therefore, the obtained LOD results confirmed that the methods employed by the laboratories were suitable for the study.

The translation of the questionnaire from English into the languages of participating countries may have led to errors or misunderstandings. However necessary, the adjustment of models for farm type and country may have explained some of the variation in HEV risk between farms and possibly lowered statistical power to detect further biosecurity measures. Finally, missing information on training or education of farm workers, specific working routines, or if any kind of biosecurity protocol was currently applied on farm may also have affected model results.

In conclusion, the prevalence of HEV in European pig farms represents a significant challenge, yet the establishment of a universally reliable control strategy remains elusive. Study results indicate that external biosecurity measures, such as the implementation of quarantine areas, and the regular evaluation of cleaning procedures through bacterial growth tests, could form cornerstones of an effective biosecurity protocol. However, more research is needed to validate findings and to better understand the infection dynamics of HEV. Finally, findings need to be disseminated to and accepted by the farming community to improve biosecurity standards on a national level and to mitigate the impact of HEV on animal and human health.

## Data availability statement

The raw data supporting the conclusions of this article will be made available by the authors, without undue reservation.

## Ethics statement

Ethical approval was not required for the studies involving animals in accordance with the local legislation and institutional requirements because no procedures were performed that fall within the definition of animal experimentation and thereby the study was exempt from ethical evaluation for use of animals in research. Sampling from fecal deposits on the ground was deemed outside of the Animal (Scientific Procedures) Act 1986. Written informed consent was obtained from the owners for the participation of their animals in this study.

## Author contributions

TD: Data curation, Formal analysis, Visualization, Writing – original draft, Writing – review & editing. MM: Conceptualization, Investigation, Methodology, Writing – review & editing. RS: Conceptualization, Data curation, Funding acquisition, Investigation, Methodology, Project administration, Supervision, Writing – review & editing. TT: Conceptualization, Investigation, Methodology, Supervision, Writing – review & editing. ID: Investigation, Methodology, Writing – original draft, Writing – review & editing. RJ: Conceptualization, Methodology, Supervision, Writing – review & editing. EP: Investigation, Methodology, Writing – review & editing. GK-V: Methodology, Writing – review & editing, Data curation, Investigation. ES: Conceptualization, Investigation, Methodology, Resources, Writing – review & editing. CP: Conceptualization, Investigation, Methodology, Resources, Writing – review & editing. GA: Methodology, Project administration, Writing – review & editing. HM: Conceptualization, Investigation, Resources, Writing – review & editing. NA: Investigation, Writing – review & editing. GI: Data curation, Investigation, Methodology, Visualization, Writing – review & editing. JŻ: Investigation, Writing – review & editing. AD: Investigation, Writing – review & editing, Methodology. GLA: Investigation, Writing – review & editing. DD'A: Formal analysis, Investigation, Writing – review & editing. SS: Investigation, Writing – review & editing. NB: Writing – review & editing, Investigation. EB: Conceptualization, Funding acquisition, Investigation, Project administration, Software, Supervision, Writing – review & editing.
