# Corrigendum: Biosecurity measures to control hepatitis E virus on European pig farms

**DOI:** 10.3389/fvets.2024.1441544

**Published:** 2024-06-25

**Authors:** Tamino Dubbert, Marina Meester, Richard Piers Smith, Tijs J. Tobias, Ilaria Di Bartolo, Reimar Johne, Enrico Pavoni, Gergana Krumova-Valcheva, Elena Lucia Sassu, Christopher Prigge, Giuseppe Aprea, Hannah May, Nadine Althof, Giovanni Ianiro, Jacek Żmudzki, Albena Dimitrova, Giovanni Loris Alborali, Daniela D'Angelantonio, Silvia Scattolini, Noemi Battistelli, Elke Burow

**Affiliations:** ^1^Department of Biological Safety, German Federal Institute for Risk Assessment (BfR), Berlin, Germany; ^2^Department of Population Health Sciences, Faculty of Veterinary Medicine, Utrecht University (UU), Utrecht, Netherlands; ^3^Department of Epidemiological Sciences, Animal and Plant Health Agency (APHA) - Weybridge, Surrey, United Kingdom; ^4^Department of Food Safety, Nutrition and Veterinary Public Health, Istituto Superiore di Sanità (ISS), Rome, Italy; ^5^Food Safety Department, Experimental Zooprophylactic Institute of Lombardy and Emilia Romagna (IZSLER), Brescia, Italy; ^6^National Food Safety Center, National Diagnostic and Research Veterinary Medical Institute (NDRVMI), Sofia, Bulgaria; ^7^Institute for Veterinary Disease Control, Austrian Agency for Health and Food Safety (AGES), Mödling, Austria; ^8^Department of Food Safety, Experimental Zooprophylactic Institute of Abruzzo and Molise ‘G. Caporale' (IZS), Teramo, Italy; ^9^Department of Swine Diseases, National Veterinary Research Institute (PIWet), Puławy, Poland; ^10^Department for Rural Development and Agriculture, Ministry of Agriculture, Environment and Climate Protection of the State of Brandenburg (MLUK), Potsdam, Germany

**Keywords:** hepatitis-E-virus, HEV, biosecurity measures, pig production, BIOPIGEE, One Health, OH

In the published article, there was an error in the author list, and author Christopher Prigge (initials: CP) was erroneously excluded. The corrected author list appears below.

Tamino Dubbert, Marina Meester, Richard Piers Smith, Tijs J. Tobias, Ilaria Di Bartolo, Reimar Johne, Enrico Pavoni, Gergana Krumova-Valcheva, Elena Lucia Sassu, Christopher Prigge, Giuseppe Aprea, Hannah May, Nadine Althof, Giovanni Ianiro, Jacek Zmudzki, Albena Dimitrova, Giovanni Loris Alborali, Daniela D'Angelantonio, Silvia Scattolini, Noemi Battistelli and Elke Burow

In the published article, there was an error. As a new author's name has been added to the list, his initials has to be added in the Author Contributions.

A correction has been made to Author contributions. This sentence previously stated:

“TD: Data curation, Formal analysis, Visualization, Writing – original draft, Writing – review & editing. MM: Conceptualization, Investigation, Methodology, Writing – review & editing. RS: Conceptualization, Data curation, Funding acquisition, Investigation, Methodology, Project administration, Supervision, Writing – review & editing. TT: Conceptualization, Investigation, Methodology, Supervision, Writing – review & editing. ID: Investigation, Methodology, Writing – original draft, Writing – review & editing. RJ: Conceptualization, Methodology, Supervision, Writing – review & editing. EP: Investigation, Methodology, Writing – review & editing. GK-V: Methodology, Writing – review & editing, Data curation, Investigation. ES: Conceptualization, Investigation, Methodology, Resources, Writing – review & editing. GA: Methodology, Project administration, Writing – review & editing. HM: Conceptualization, Investigation, Resources, Writing – review & editing. NA: Investigation, Writing – review & editing. GI: Data curation, Investigation, Methodology, Visualization, Writing – review & editing. JŻ: Investigation, Writing – review & editing. AD: Investigation, Writing – review & editing, Methodology. GLA: Investigation, Writing – review & editing. DD'A: Formal analysis, Investigation, Writing – review & editing. SS: Investigation, Writing – review & editing. NB: Writing – review & editing, Investigation. EB: Conceptualization, Funding acquisition, Investigation, Project administration, Software, Supervision, Writing – review & editing”.

The corrected sentence appears below:

“TD: Data curation, Formal analysis, Visualization, Writing – original draft, Writing – review & editing. MM: Conceptualization, Investigation, Methodology, Writing – review & editing. RS: Conceptualization, Data curation, Funding acquisition, Investigation, Methodology, Project administration, Supervision, Writing – review & editing. TT: Conceptualization, Investigation, Methodology, Supervision, Writing – review & editing. ID: Investigation, Methodology, Writing – original draft, Writing – review & editing. RJ: Conceptualization, Methodology, Supervision, Writing – review & editing. EP: Investigation, Methodology, Writing – review & editing. GK-V: Methodology, Writing – review & editing, Data curation, Investigation. ES: Conceptualization, Investigation, Methodology, Resources, Writing – review & editing. CP: Conceptualization, Investigation, Methodology, Resources, Writing – review & editing. GA: Methodology, Project administration, Writing – review & editing. HM: Conceptualization, Investigation, Resources, Writing – review & editing. NA: Investigation, Writing – review & editing. GI: Data curation, Investigation, Methodology, Visualization, Writing – review & editing. JŻ: Investigation, Writing – review & editing. AD: Investigation, Writing – review & editing, Methodology. GLA: Investigation, Writing – review & editing. DD'A: Formal analysis, Investigation, Writing – review & editing. SS: Investigation, Writing – review & editing. NB: Writing – review & editing, Investigation. EB: Conceptualization, Funding acquisition, Investigation, Project administration, Software, Supervision, Writing – review & editing”.

The authors apologize for this error and state that this does not change the scientific conclusions of the article in any way. The original article has been updated.

