# Supplementary material for: Biosecurity measures to control hepatitis E virus on European pig farms
Source: Front Vet Sci. 2024 Feb 14;11:1328284. doi: 10.3389/fvets.2024.1328284 (PMC11231669; doi:10.3389/fvets.2024.1328284)
Supplement: Supplementary file 3 [file Table_3.DOCX]

# Supplementary Materials

Supplementary Table 1: Samples per farm types among all farms

| **Farm type** | **Positive** | **Total** | **Mean_pos** | **Mean_perc** |
| --- | --- | --- | --- | --- |
| Farrow-to-finish | 294 | 2269 | 6 | 13 |
| Breeding | 36 | 792 | 7.5 | 4.5 |
| Fattening | 388 | 1328 | 0.5 | 29.2 |
| **Total** | **718** | **4389** | **3.1** | **16.4** |

Supplementary Table 2: Samples per farm types only among positive farms

| **Farm type** | **Positive** | **Total** | **Mean_pos** | **Mean_perc** |
| --- | --- | --- | --- | --- |
| Farrow-to-finish | 294 | 1244 | 10.9 | 23.6 |
| Breeding | 36 | 204 | 29.4 | 17.6 |
| Fattening | 388 | 898 | 0.7 | 43.2 |
| **Total** | **718** | **2346** | **5.7** | **30.6** |

Supplementary Table 3: Proportion of positive farms among all farms per farm type

| **Farm type** | **Positive** | **Total** | **Percent** |
| --- | --- | --- | --- |
| Farrow-to-finish | 66 | 119 | 55.5 |
| Breeding | 10 | 39 | 25.6 |
| Fattening | 51 | 73 | 69.9 |
| **Total** | **127** | **231** | **55** |

Supplementary Table 4: Sample distribution among all outdoor farms and only among positive outdoor farms

| **Outdoor farms** | **Positive** | **Total** | **Mean_pos** | **Mean_perc** |
| --- | --- | --- | --- | --- |
| All (6) | 7 | 119 | 1.2 | 5.9 |
| HEV-positive (2) | 7 | 39 | 3.5 | 17.9 |

Supplementary Table 5: Farms with higher HEV risk among all farms per farm type

| **Farm type** | **Higher risk** | **Total** | **Percent** |
| --- | --- | --- | --- |
| Farrow-to-finish | 34 | 119 | 28.6 |
| Breeding | 3 | 39 | 7.7 |
| Fattening | 35 | 73 | 47.9 |
| **Total** | **72** | **231** | **31.2** |

**Samples per farm**

Regarding samples of pigs from all production stages and from all farms including serum samples from NL, between 4 and 24 samples were delivered per farm with a median of 20 (IQR: 0) and a mean average of 19 samples per farm. Positive samples per farm (i.e. gilts, dry sows, and fattener samples of all ages) ranged from 0 to 20 positive samples with a median average of 1 (IQR: 5) and a mean average of 3.1 positive samples per farm.

Regarding the fattener samples only from farrow-to-finish and fattening farms including serum samples from NL farrow-to-finish and fattening farms, farms had between 4 and 21 samples, with a median of 10 (IQR: 10) and a mean average of 13.2 fattener samples. Positive fattener samples ranged from 0 to 20 per farm, with a median of 1 (IQR: 6) and a mean average of 3.5 positive fattener samples.

**Pig samples**

Samples from fattener pigs were most frequently HEV-positive, followed by samples from gilts and dry sows, with 653 (26%), 48 (5%), and 17 (2%) positive samples. Overall, 16.4% of all pig samples were HEV positive.

**Calculating fattener HEV risk**

According to the sampling scheme, ten samples from farrow-to-finish farms were from fatteners (50%). Additionally, the number of samples from NL farms varied, as they were produced from another study. Therefore, when calculating the fattener risk score, the HEV risk of farrow-to-finish and some NL farms was only based on 10 samples or fewer, compared to 20 fattener samples from fattener farms. When only fattener samples were considered, 120 farms (63.83%) had 10 or fewer fattener samples, while 68 farms (36.17%) had 11 or more fattener samples. A chi-square test showed, that farms with 10 or fewer fattener samples were not significantly more likely to be in the lower risk category than farms with more than 10 fattener samples (OR 1.32, 95% CI: 0.69-2.5, p = 0.373).

**Univariable results**

Variables independently associated with higher HEV risk based on all samples in univariable models, which controlled for country and farm type, were: ‘always cleaning footwear when moving between barn sections’, ‘48 to 72h pig contact free period for external people required before entering the barn’ (only significant when including Czech farms), ‘not testing or treating purchased pig feed for *Salmonella* contamination’, ‘cleaning of feeding pipelines at least once a year’, ‘cleaning and disinfection of feeding pipelines at least once a year’, and ‘a pest control program against rodents carried out by the farmer’ (Supplementary Table 6).

Variables independently associated with lower HEV risk, were: ‘five or fewer people in charge of the pigs’, ‘hygienograms used in the breeding or weaning area after cleaning’, and ‘pest control program carried out against wild birds by a professional company’ (Supplementary Table 6).

Variables independently associated with higher HEV risk based only on fattener samples in models which controlled for country and farm type, were: ‘48 to 72h pig contact free period required for external people before entering the barn’ (only significant when including Czech farms), ‘not testing or treating purchased pig feed for *Salmonella* contamination’, ‘cleaning of feeding pipelines at least once a year’, ‘no pit below (slatted) flooring in the quarantine area’, and ‘no other livestock species present on farm’ (Supplementary Table 7).

Variables independently associated with lower HEV risk were: ‘five or fewer people in charge of the pigs’, ‘hygienograms used in the breeding, farrowing, or weaning areas after cleaning’, and ‘pest control program carried out against wild birds by a professional company’ (Supplementary Table 7).

Supplementary Table 6: Results of significant predictors of HEV based on all samples in univariable logistic regression analyses adjusted for farm type and country

| Question | Labels | Higher.HEV.Risk.n(%) | OR | 95%.CI.[LL,.UL] | P |
| --- | --- | --- | --- | --- | --- |
| Is footwear always cleaned between barn sections or when moving to another production stage (e.g. from weaners to fatteners)? (Q18_4) | No | 47 (27 %) | 1 |  |  |
|  | Yes | 15 (41 %) | 2.3 | 1, 5.2 | 0.046 |
|  | None | 10 (43 %) | 1.2 | 0.4, 3.5 | 0.767 |
| How many internal persons are in charge of taking care of the pigs? (Q19x2) | Six to one hundred | 24 (39 %) | 1 |  |  |
|  | One to five | 48 (28 %) | 0.2 | 0.1, 0.6 | 0.003 |
| Is there a pig-contact free period of >12h before any external people are allowed to enter the barn? (Q21) | No | 42 (31 %) | 1 |  |  |
|  | Yes. <12h | 2 (20 %) | 1.4 | 0.2, 7.8 | 0.736 |
|  | Yes. 12 to 24h | 5 (31 %) | 1.1 | 0.3, 3.8 | 0.88 |
|  | Yes. 24 to 48h | 6 (22 %) | 1.3 | 0.4, 4.2 | 0.691 |
|  | Yes. 48 to 72h | 15 (50 %) | 5.9 | 1.7, 20.7 | 0.006 |
|  | Yes. >72h | 2 (14 %) | 0.5 | 0.1, 2.8 | 0.435 |
| Is purchased feed NOT always tested for or treated against Salmonella contamination? (Q38_2_1) | No | 42 (29 %) | 1 |  |  |
|  | Yes | 30 (35 %) | 2.4 | 1.1, 5.2 | 0.03 |
| Do the feeding pipelines get cleaned at least once a year? (Q42_2) | No | 28 (30 %) | 1 |  |  |
|  | Yes. cleaned | 21 (30 %) | 2.6 | 1, 6.3 | 0.039 |
|  | Yes. cleaned & disinfected | 21 (34 %) | 3.6 | 1.3, 10.3 | 0.015 |
|  | No answer | 2 (29 %) | 4.5 | 0.6, 33.3 | 0.144 |
| Is a pest control program against rodents carried out by the farmer? (Q67_2) | No | 1 (8 %) | 1 |  |  |
|  | Yes | 71 (33 %) | 12.3 | 1.4, 104.4 | 0.022 |
| Is a pest control program against wild birds carried out by a professional company? (Q68_3) | No | 61 (35 %) | 1 |  |  |
|  | Yes | 10 (21 %) | 0.2 | 0.1, 0.6 | 0.008 |
|  | N/A. missing | 1 (8 %) | 0.1 | 0, 0.7 | 0.026 |
| Efficacy check of cleaning procedure in breeding area between batches (Q55_1_8) | No | 31 (25 %) | 1 |  |  |
|  | Yes | 5 (17 %) | 0.3 | 0.1, 0.9 | 0.039 |
|  | None | 36 (48 %) | 4 | 0.1, 120.8 | 0.427 |
| Efficacy check of cleaning procedure in weaning area between batches (Q55_3_8) | No | 35 (27 %) | 1 |  |  |
|  | Yes | 5 (17 %) | 0.3 | 0.1, 1 | 0.045 |
|  | None | 32 (45 %) | 1 | 0.3, 3.3 | 0.976 |

Supplementary Table 7: Results of significant predictors of HEV based on all samples in univariable logistic regression analyses adjusted for farm type and country

| Question | Labels | Higher.HEV.Risk.n(%) | OR | 95%.CI.[LL,.UL] | P |
| --- | --- | --- | --- | --- | --- |
| How many internal persons are in charge of taking care of the pigs? (Q19x2) | Six to one hundred | 24 (51 %) | 1 |  |  |
|  | One to five | 53 (38 %) | 0.2 | 0.1, 0.6 | 0.006 |
| Is there a pig-contact free period of >12h before any external people are allowed to enter the barn? (Q21) | No | 45 (41 %) | 1 |  |  |
|  | Yes, <12h | 2 (33 %) | 1.2 | 0.2, 7.5 | 0.827 |
|  | Yes, 12 to 24h | 5 (36 %) | 0.9 | 0.3, 3 | 0.829 |
|  | Yes, 24 to 48h | 7 (37 %) | 1.1 | 0.3, 3.7 | 0.841 |
|  | Yes, 48 to 72h | 14 (58 %) | 3.8 | 1, 13.9 | 0.049 |
|  | Yes, >72h | 4 (29 %) | 1 | 0.2, 3.9 | 0.966 |
| Is all feed always tested for or treated against Salmonella contamination? | No | 42 (36 %) | 1 |  |  |
|  | Yes | 35 (49 %) | 2.8 | 1.3, 6.2 | 0.011 |
| Do the feeding pipelines get cleaned at least once a year? (Q42_2) | No | 32 (41 %) | 1 |  |  |
|  | Yes, cleaned | 24 (44 %) | 2.6 | 1.1, 6.4 | 0.034 |
|  | Yes, cleaned & disinfected | 19 (38 %) | 2.6 | 0.9, 7.5 | 0.07 |
|  | No answer | 2 (40 %) | 2.9 | 0.4, 24 | 0.314 |
| Efficacy check of cleaning procedure in breeding area between batches (Q55_1_8) | No | 36 (41 %) | 1 |  |  |
|  | Yes | 5 (19 %) | 0.2 | 0.1, 0.8 | 0.019 |
|  | None | 36 (49 %) | 1929308.8 | 0, Inf | 0.987 |
| Efficacy check of cleaning procedure in farrowing area between batches (Q55_2_8) | No | 37 (42 %) | 1 |  |  |
|  | Yes | 5 (19 %) | 0.2 | 0.1, 0.8 | 0.018 |
| Efficacy check of cleaning procedure in weaning area between batches (Q55_4_8) | No | 41 (42 %) | 1 |  |  |
|  | Yes | 5 (19 %) | 0.2 | 0.1, 0.8 | 0.018 |
|  | None | 31 (48 %) | 1 | 0.2, 3.8 | 0.946 |
| Is the pit below slatted flooring emptied between two batches per production stage in the quarantine area? (Q59_5) | Yes | 6 (20 %) | 1 |  |  |
|  | No | 5 (29 %) | 1.4 | 0.3, 5.9 | 0.637 |
|  | No pit present | 9 (45 %) | 3.9 | 1, 15.2 | 0.047 |
|  | Not Present at farm | 57 (47 %) | 3.6 | 1.2, 10.4 | 0.021 |
| Is a pest control program against wild birds carried out by a professional company? (Q68_3) | No | 67 (49 %) | 1 |  |  |
|  | Yes | 10 (24 %) | 0.2 | 0, 0.6 | 0.006 |
|  | N/A, missing | 0 (0 %) | Predicts failure perfectly |  |  |
| Are there other livestock species present on the farm? (Q72) | Yes | 12 (30 %) | 1 |  |  |
|  | No | 65 (44 %) | 2.4 | 1, 5.7 | 0.047 |

Supplementary Table 8: Results of sensitivity analysis of multivariable risk factor model with HEV outcome based on all samples and farms of country ‘NL’ removed

| Question | Levels | Higher.HEV.Risk.n(%) | OR | 95%.CI.[LL,.UL] | P-value |
| --- | --- | --- | --- | --- | --- |
| Number of people in charge of the pigs | 6+ | 22 (36.7) | 1 |  |  |
|  | 1-5 | 38 (25.2) | 0.12 | 0.03, 0.38 | 0.001 |
| Is an efficacy check with a hygienogram part of the cleaning procedures in the breeding area? | No | 28 (23.3) | 1 |  |  |
|  | Yes | 5 (16.7) | 0.07 | 0.01, 0.33 | 0.002 |
|  | N/A | 27 (44.3) | 78.39 | Inf, Inf | 0.999 |
| Is a quarantine area present at your farm? | No | 13 (26.5) | 1 |  |  |
|  | Yes | 21 (19.6) | 0.29 | 0.09, 0.89 | 0.033 |
|  | N/A | 26 (47.3) | 0.61 | 0.05, 16.18 | 0.722 |
| Is downtime of at least three dayspart of the cleaning procedures in the fattening area? | No | 23 (26.1) | 1 |  |  |
|  | Yes | 35 (36.8) | 3.02 | 1.22, 7.86 | 0.019 |
|  | N/A | 2 (7.1) | 0.25 | 0.02, 3.42 | 0.289 |
| Do internal people always have to wash hands between different barn sections? | No | 42 (26.2) | 1 |  |  |
|  | Yes | 16 (37.2) | 3.47 | 1.2, 10.56 | 0.024 |
|  | N/A | 2 (25) | 0.34 | 0.04, 2.06 | 0.269 |
| Are disposable gloves worn when manipulating carcasses and/or are hands washed and disinfected after manipulating carcasses? | No | 0 (0) | 1 |  |  |
|  | Yes | 60 (29.7) | 45739333.66 | 0, Inf | 0.988 |
| Do wild birds have access to the barns? | No | 39 (27.7) | 1 |  |  |
|  | Yes | 19 (30.2) | 2.85 | 1.11, 7.61 | 0.032 |
|  | N/A | 2 (28.6) | 2.92 | 0.3, 24.3 | 0.324 |

Supplementary Table 9: Results of sensitivity analysis of multivariable risk factor model with HEV outcome based on finisher samples only and farms of country ‘NL’ removed

| Question | Levels | Higher.HEV.Risk.n(%) | OR | 95%.CI.[LL,.UL] | P-value |
| --- | --- | --- | --- | --- | --- |
| Number of people in charge of the pigs | 6+ | 22 (48.9) | 1 |  |  |
|  | 1-5 | 43 (35) | 0.13 | 0.03, 0.47 | 0.003 |
| Is purchased feed always tested for and/or treated against Salmonella contamination? | No | 35 (48.6) | 1 |  |  |
|  | Yes | 30 (31.2) | 0.34 | 0.13, 0.83 | 0.02 |
| Is an efficacy check with a hygienogram part of the cleaning procedures in the fattening area? | No | 57 (41.3) | 1 |  |  |
|  | Yes | 8 (26.7) | 0.23 | 0.06, 0.82 | 0.026 |
| Is a quarantine area present at your farm? | No | 15 (45.5) | 1 |  |  |
|  | Yes | 24 (30) | 0.25 | 0.08, 0.74 | 0.014 |
|  | N/A | 26 (47.3) | 0.41 | 0.03, 11.06 | 0.524 |
| Are pigs purchased? | No | 17 (28.8) | 1 |  |  |
|  | Yes | 48 (44) | 3.43 | 1.38, 9.09 | 0.01 |
| Are other livestock species present on the farm? | No | 57 (41.6) | 1 |  |  |
|  | Yes | 8 (25.8) | 0.25 | 0.07, 0.77 | 0.02 |
| Do wild birds have access to the barns? | No | 42 (35.9) | 1 |  |  |
|  | Yes | 21 (45.7) | 3.33 | 1.33, 8.77 | 0.012 |
|  | N/A | 2 (40) | 2.86 | 0.26, 30.95 | 0.371 |
